# All-Wales licensed premises intervention (AWLPI): a randomised controlled trial to reduce alcohol-related violence

**DOI:** 10.1186/1471-2458-14-21

**Published:** 2014-01-10

**Authors:** Simon C Moore, Claire O’Brien, Mohammed Fasihul Alam, David Cohen, Kerenza Hood, Chao Huang, Laurence Moore, Simon Murphy, Rebecca Playle, Vaseekaran Sivarajasingam, Irena Spasic, Anne Williams, Jonathan Shepherd

**Affiliations:** 1 Violence & Society Research Group, School of Dentistry, College of Biomedical and Life Sciences, Cardiff University, Cardiff CF14 4XY, UK; 2 DECIPHer, Cardiff School of Social Sciences, Cardiff University, 1-3 Museum Place, Cardiff CF10 3BD, UK; 3 South East Wales Trials Unit, Institute of Translation, Innovation, Methodology and Engagement, Cardiff University, Neuadd Meirionnydd, Heath Park, Cardiff CF14 4YS, UK; 4 School of Computer Science, Cardiff University, Queen’s Buildings, 5 The Parade, Cardiff CF24 3AA, UK; 5 Faculty of Life, Science and Education, University of South Wales, Pontypridd, CF37 1DL, UK; 6 MRC/CSO Social and Public Health Sciences Unit, University of Glasgow, 4 Lilybank Gardens, Glasgow G12 8RZ, UK

**Keywords:** Alcohol, Violence, Licensed premises, Night time economy, Health and safety

## Abstract

**Background:**

Alcohol-related violence in and in the vicinity of licensed premises continues to place a considerable burden on the United Kingdom’s (UK) health services. Robust interventions targeted at licensed premises are therefore required to reduce the costs of alcohol-related harm. Previous evaluations of interventions in licensed premises have a number of methodological limitations and none have been conducted in the UK. The aim of the trial was to determine the effectiveness of the Safety Management in Licensed Environments intervention designed to reduce alcohol-related violence in licensed premises, delivered by Environmental Health Officers, under their statutory authority to intervene in cases of violence in the workplace.

**Methods/Design:**

A national randomised controlled trial, with licensed premises as the unit of allocation. Premises were identified from all 22 Local Authorities in Wales. Eligible premises were those with identifiable violent incidents on premises, using police recorded violence data. Premises were allocated to intervention or control by optimally balancing by Environmental Health Officer capacity in each Local Authority, number of violent incidents in the 12 months leading up to the start of the project and opening hours. The primary outcome measure is the difference in frequency of violence between intervention and control premises over a 12 month follow-up period, based on a recurrent event model. The trial incorporates an embedded process evaluation to assess intervention implementation, fidelity, reach and reception, and to interpret outcome effects, as well as investigate its economic impact.

**Discussion:**

The results of the trial will be applicable to all statutory authorities directly involved with managing violence in the night time economy and will provide the first formal test of Health and Safety policy in this environment. If successful, opportunities for replication and generalisation will be considered.

**Trial registration:**

UKCRN 14077; ISRCTN78924818.

## Background

Alcohol related violence continues to place a considerable burden on health services [1]. Approximately 70% of unscheduled Accident and Emergency (A&E) attendances are alcohol related at peak times [2], with the bulk stemming from activities in the night time economy (NTE) [3,4]. Urban centres characterised by a high density of premises licensed for the on-site sale and consumption of alcohol typically produce a substantial share of all alcohol-related harm and are associated with severe intoxication and violent injury [5,6]. There is a growing literature detailing environment-specific risk factors in the on-licensed trade [4,7] and recognition that interventions that address these are urgently required [8,9]. However, the number of front-line police staff available to manage this environment is decreasing [10] and in the UK there is growing reliance on unevaluated industry-sponsored schemes. There is therefore a need for robust, formally evaluated interventions that can be routinely adopted by partners involved with managing the NTE. Previous evaluations of premises level (PL) interventions have a number of methodological limitations and none have been conducted in the UK.

There have been two systematic reviews in this area [4,11]. The earlier review focussed on server training interventions and concluded that research in the context of the NTE should be broadened to develop interventions that address more complex causal pathways and multiple risk factors across the full socio-ecological environment [11]. A more recent review, [4] assessed broader approaches to prevention that included responsible beverage service (RBS) training (n = 6), enhanced licensing regulation enforcement (n = 2), multi-level interventions (n = 6), licensee accords (n = 2) and a risk-focused consultation (n = 1). Only five randomised controlled trials (RCT) were identified and these were subject to a number of shortcomings including (i) considerable variation in and poorly defined outcome measures meaning studies could not be compared, (ii) follow-up periods were decided ad hoc and did not consider intervention sustainability, (iii) economic evaluations were not included, (iv) studies often relied on inappropriate control groups, (v) many failed to achieve random allocation, and (vi) participants or evaluators were not blind to study conditions. The review concluded that, while interventions that address multiple risk factors and that are designed and implemented by multi-agency and community partnerships have the potential to be effective, there is little rigorous evidence of effectiveness. It therefore recommended further development and piloting phases for complex interventions addressing multiple risk factors as a pre-requisite for their rigorous evaluation and any subsequent implementation.

The current intervention builds on earlier work that identified causative factors for violence that might be realistically targeted by interventions that are both theoretically and practically robust [12,13]. A subsequent project designed specifically to examine the case for a RCT of a multi-risk PL intervention [14] was completed. The project developed and implemented appropriate intervention content, tested its feasibility and acceptability, identified causal pathways linking intervention to violence reduction and established the feasibility of key aspects of RCT design including outcome measures, recruitment and retention. Outputs from this project were the first to rigorously test all aspects of a multi-level PL intervention in a UK context. Key findings were: (i) an enhanced multiple risk audit approach can successfully identity appropriate targets and approaches to prevention; (ii) the engagement of licensed premises and the efficacy of the intervention were maximised when implemented by statutory authorities [14]; and (iii) police recorded data on violent incidents were a valid measure of harm and sensitive to change at the PL [15]. In addition, the nested process evaluation (PE) indicated that it was necessary to engage staff across the premises hierarchy, from servers through premises managers and, where appropriate, regional managers [16] to ensure intervention receptivity.

**Figure 1:**
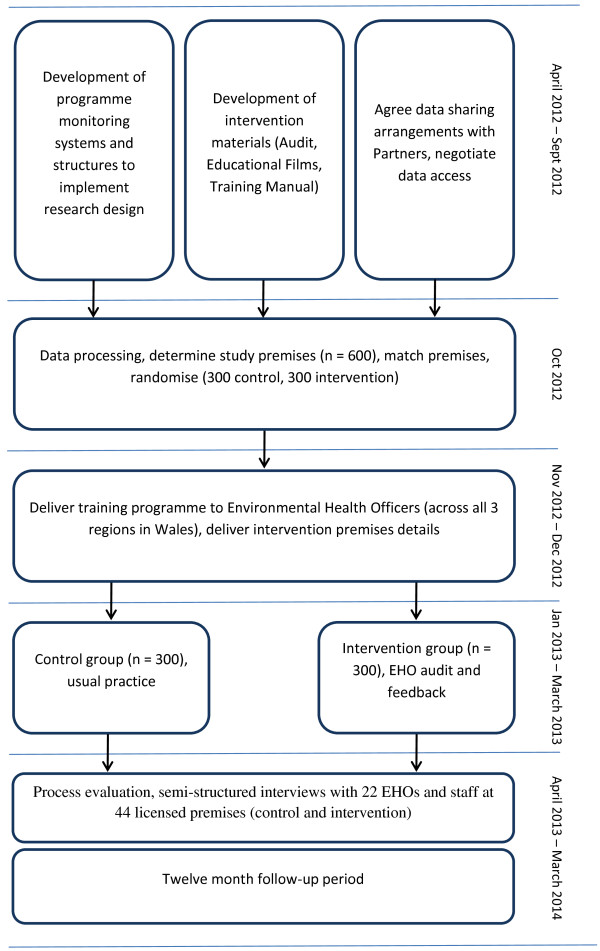
Trial design.

The theoretical basis of the intervention (SMILE: Safety Management In Licensed Environments) evaluated in the current trial is that reducing known risk factors [17] within premises and their immediate environment will either directly or indirectly reduce alcohol misuse and violence and that current legislation (2003 Licensing Act, Health and Safety at Work Act 1974) provides a framework for the delivery of a risk audit intervention. Taken together, this motivates an intervention process whereby at-risk premises are first identified using police recorded violence data. Information is then shared with Environmental Health Officers (EHO) who audit and identify premises-specific risks. The intervention itself was initially developed from formative developmental studies and was further refined in an additional development phase (see Figure 1). The aim of this additional development stage was to situate the theoretical and empirical motivations for the intervention within EHO statutory authority. EHOs are not able to enforce the UK 2003 Licensing Act which is primarily focused on the sale and consumption of alcohol; however they do have available legislation that covers the operation of businesses with a view to harm reduction, including violence in the workplace. For example, businesses with five or more employees are required to undertake a formal risk assessment that should be written and available to staff. We therefore sought to identify what risks within premises might reasonably be enforced by EHOs under the Health and Safety at Work Act and developed intervention materials and guidance to EHOs to facilitate their work in these areas.

In addition, more general Health and Safety guidance for EHOs develops the use of instruments such as the Risk Control Indicator (RCI) [18]. This instrument is used to describe and summarise risk in specific areas of operation and we therefore used existing templates to develop RCI metrics for use in premises. The group responsible for this development stage included senior EHOs, researchers and experts in areas relating to the NTE and the licensed trade generally. This development stage further fed into the creation of an intervention website designed for premises staff and contained information relevant to harm minimisation and their duties in that respect. The website contained videos that were amenable to view on a range of platforms (including smartphones and computers), documentation, and template forms that could be used as a basis for, for example, health and safety checks. The overall theme of the site was to provide positive and accessible messages on the advantages of a safe premises.

## Methods/Design

### Trial aims

The primary aim of the AWLPI trial is to determine the effectiveness of the SMILE intervention to reduce police recorded violence in licensed premises. Secondary aims include:

1. To determine whether the impact of the intervention changes over a twelve month follow-up period

2. To identify the costs associated with SMILE and evaluate cost effectiveness

3. To assess whether the fidelity of SMILE is maintained across Local Authorities (LAs)

4. To consider the relationship between outcomes and intervention reach, dose and receipt

5. To develop a revised logic model of the intervention

6. To determine the optimal format of the risk-led PL intervention for delivery by EHOs

### Trial design

Figure 1 provides a summary of the trial, a randomised controlled trial with embedded process and economic evaluations of the intervention designed to reduce alcohol-related violence in licensed premises across Wales.

### Ethical considerations

The trial received ethical approval from Cardiff University Dental School Research Ethics Committee (reference: 12/08).

### Study population

The study population is comprised of licensed premises that are based within the 22 LAs in Wales. To be eligible, premises must either be a public house, night club, or hotel with a public bar that has had one or more violent incidents (including Section 18/20, Section 47, common assault, affray, assault of a police officer) recorded by police in the twelve month period between May 2011 to April 2012. Other premises that are licensed for the sale of alcohol such as cafes, restaurants and entertainment venues (e.g. sports facilities, concert halls) are excluded.

### Sample size

A previous exploratory trial [19] suggested an overall group size of 272 premises provided a power of 90% to detect a 10% reduction in the failure rate (a day in which one or more violent incidents occurred) at a significance level of 0.05. Attrition is not a factor in this trial as EHOs have a statutory authority to enter premises and therefore adjustments for withdrawals are not applicable. Temporary and permanent suspensions of premises licenses are valid outcomes as they represent a form of intervention and are therefore accounted for in the group size. A 12 month follow-up period was chosen as the earlier exploratory trial suggested this offered the most likely opportunity to detect a significant effect in a full trial and could control for annualised cyclical variation within premises. As premises may appear in the police 12 month violence data but may have ceased trading between detection and the start of the project the total initial sample size was adjusted to 600.

### Recruitment

Licensed premises were identified by searching police incident data and identifying those premises in which violent incidents were detected in the period May 2011 to April 2012. Police data provides the best way of measuring violence in licensed premises as they are routinely available across the study area. Although a proportion of violent incidents are not reported to the police, these data remain the most detailed and accurate records of incidents at the premises level. Other data sources such as Emergency Department attendance data are not universally available. Police incident data are broadly consistent across the 22 LAs taking part in the trial. In order to access police data, data sharing agreements were prepared and signed between Cardiff University and all four police forces in Wales. The agreements cover the period from May 2011 to the end of the trial follow-up period (April 2014). Data containing all violence against the person incidents were encrypted and anonymised before being given to members of the research team for screening. Incidents inside and in the immediate vicinity of licensed premises were identified from the police data based on the inclusion and exclusion criteria listed above. Premises receive the intervention as part of usual practice and as such, premises do not consent to participate in the trial and so withdrawal and loss to follow-up from the intervention phase of the trial is not possible.

### Randomisation

Premises are stratified by LA, licensable hours (up to 11 pm, open beyond 11 pm), and number of violent incidents in the twelve month period between May 2011 to April 2012 and randomly allocated into control or intervention group. Optimal allocation was used to carry out the randomisation [20], which was carried out by an independent statistician within the South East Wales Trials Unit (SEWTU). The randomised sample of 600 licensed premises comprises the intention to treat (ITT) population.

### Closure and replacement premises

Information on premises closure in the intervention arm will be provided by the EHO’s during the intervention period. In the event of premises closure before the intervention phase begins, it will be replaced with a premises randomly selected from a list of any remaining premises in that LA, matched by strata. In the event that no replacement premises is available in that strata no replacement occurs. Replacement premises will also be included in a sensitivity analysis. Potential differential rates of pre**-**intervention closures due to differences in data collection methodology between arms will be examined and explored in sensitivity analyses. In both the intervention and control arms after the intervention period, closure will be determined by telephone by research team staff. Additional replacement premises will also be included in a sensitivity analysis. Premises in the intervention arm that did not cooperate with EHOs or could not be accessed by EHOs in the allotted intervention period will be included in ITT analyses.

### Trial intervention

The intervention is a premises-level risk-audit (described below) that is designed to identify those areas of operation that contribute to alcohol-related violence. After the risk-audit is complete, feedback and advice are given to the Designated Premises Supervisor (DPS) in order for improvements (where applicable) to be made to premises operation. The intervention is delivered by EHOs as they have a history of enforcement and partnership working and are trained to deliver interventions and advice to small and medium sized businesses. Furthermore, EHOs have available statutory powers that allow their entry to premises and they are able to enforce change in the interest of public health and safety.

In order to enhance intervention fidelity, EHOs who deliver the intervention attended one of three training workshops, where the rationale for the intervention, the use of the audit tool, related materials including a website designed to support change in premises and EHO training materials were explained. The intervention is conducted in all 22 LAs in Wales, UK, and details of premises to be audited and the number of premises to audit per LA (see Table 1 for details) were given to EHOs at the beginning of the intervention period. Details of premises randomised to the control arm of the trial are not disclosed to EHOs and these premises therefore receive usual treatment. The usual treatment that premises receive from EHOs relating to violence is minimal; EHOs do not usually visit LP’s following violent incidents as part of normal practice.

**Table 1:** Demographic data for each Local Authority in Wales

**Local Authority (LA)**	Population*	Population density rate (no. per hectare)*	Violent incidents**	**Audits to complete**
Blaenau Gwent	69,814	6.4	40	6
Bridgend	139,178	5.6	69	13
Caerphilly	178,806	6.4	116	19
Cardiff	346,090	24.7	239	39
Carmarthenshire	183,777	0.8	124	18
Ceredigion	75,922	0.4	37	7
Conwy	115,228	1	128	10
Denbighshire	93,734	1.1	135	9
Flintshire	152,506	3.5	99	16
Gwynedd	121,874	0.5	165	12
Isle of Anglesey	69,751	1	54	7
Merthyr Tydfil	58,802	5.3	18	5
Monmouthshire	91,323	1.1	57	8
Neath Port Talbot	139,812	3.2	48	12
Newport	145,736	7.6	175	15
Pembrokeshire	122,439	0.8	96	11
Powys	132,976	0.3	68	12
Rhondda Cynon Taf	234,410	5.5	78	25
Swansea	239,023	6.3	178	26
The Vale of Glamorgan	126,336	3.8	25	8
Torfaen	91,075	7.2	57	9
Wrexham	134,844	2.7	230	13
Total	3,063,456	4.33	2236	300

*figures from 2011 census.

**data based on 837 licensed premises eligible for inclusion in the AWLPI trial, data obtained from police incident reports between May 2011 - April 2012).

### Risk-audit

The intervention is a Risk Led Intervention (RLI) developed from literature documenting features of premises that contribute to harm [21-23]. These interventions involve an initial risk-audit followed by an action plan which is given to the DPS. If the action plan is adopted, risks that promote harm within the premises will have been addressed and a reduction in alcohol-related harm is expected. Action plans require premises to make changes to operating procedures (e.g. reducing capacity, changing how security staff are deployed, checking patrons’ age at the door), improve staff training, as well as covering aspects of the internal and external environment (such as improving surveillance).

The RLI is made up of three components: first, EHOs audit intervention premises to identify areas that might increase the risk of violence, with findings recorded on a risk-audit form (see list of Information recorded by EHO’s on the risk-audit form(s)). For each area of premises operation covered by the risk-audit, EHOs record as much information as possible before giving an RCI score which corresponds with the perceived level of risk. Second, EHOs then record what further action (if any) is required at the premises. If risks are identified EHOs could respond in one or more of the following ways: 1) advise premises to make changes, i.e. verbally or by informal letter; 2) formally require changes to be made, i.e. by serving an improvement notice; 3) refer premises to police and LA licensing officers (who are able to place conditions on premises licenses); 4) a prohibition notice could be served to close the premises without notice if risks present a clear and immediate danger to the public. EHOs will conduct a second audit in premises where further action is required to assess whether the required changes have been made (and enforce where appropriate). The second audit will focus only on areas of risk identified in the initial audit. Depending upon the severity of the risk identified in the initial audit, the second audit will take place either one month (for serious risks) or three months (less serious risks) later, consistent with EHO usual practice. Following the initial risk-audit, EHOs will encourage intervention premises DPS and bar staff to access web-based training and instructional materials designed to engage them in harm reduction practices.

Information recorded by EHOs on the risk-audit form(s)

Audit date*

Audit start and end times*

EHO information (name, contact information, salary)*

Premises information (e.g. number of staff, food served, live music, etc.)

Areas of premises operation to assess

– Record checks (e.g. safety policy, risk assessments)*

– Visibility and lighting*

– Health and safety observation and checks*

– Surveillance*

– Noise and communication*

– Risk planning*

– Door management*

– Managing people*

– Staff training*

– Incident reporting (to RIDDOR, http://www.hse.gov.uk/riddor/, archived at http://www.webcitation.org/6L31Hvs03)*

– Glassware policy*

Questions for servers to assess the extent that premises policy reaches frontline staff

EHOs confidence in premises management*

Action taken by EHO*

* Indicates areas covered in the follow-up audit, if applicable.

### Web-based training and instructional materials

The intervention website contains information about harm reduction practices in licensed premises and provides guidance documents that can be downloaded and used by premises staff. The website also contains training and educational films, as well as a due diligence quiz, that are designed to provide instruction on how premises staff can reduce excessive alcohol consumption and violence. The website is available in English and Welsh [24].

Training and educational films are designed to increase knowledge of policies and practices that prevent and reduce excessive alcohol consumption and violence. The training and educational films offer guidance in the following areas: premises environment, security, crowding and how to de-escalate fractious encounters between customers. Films were also distributed by EHOs on DVD to cover premises without reliable internet access.

The due-diligence quiz comprises twenty-five questions that assess understanding and knowledge gained through viewing training films. Members of premises staff answering ≥50% of questions correctly receive a certificate of achievement that can be displayed in premises.

Reference materials were also provided. These are downloadable guidance documents, document templates, and posters that collectively aim to help premises staff reduce alcohol-related violence in their premises.

Business cards are used to advertise the website address. The business cards are given to premises staff by EHOs after the initial audit and are available in English and Welsh.

### Process evaluation

The trial includes an embedded process evaluation that assesses programme implementation and therefore facilitates interpretation of outcome effects. A critical realistic approach [25] was used to elucidate what works best, for whom, in what context, with particular focus on intervention reach, acceptability, implementation, fidelity and sustainability. The process evaluation was concerned with six core research aims:

1. Understand the implementation and context of the intervention

2. Assess the fidelity of the intervention and the adaptation required to integrate the intervention within routine practice and different Local Authorities

3. Assess the reach and dose delivered of the intervention

4. Assess receipt and acceptability of the intervention

5. To compare traditional practice in licensed premises with the intervention

6. Refine the intervention and construct a logic model

In order to address the research aims, semi-structured interviews and focus groups will be carried out with senior industry executives, senior EHOs, EHOs, and licensed premises staff. Participation in the process evaluation is voluntary and participants must give written consent to take part. Consent may be withdrawn at any point; however, data provided prior to withdrawal will be used in the analysis unless requested otherwise. An overview of the process evaluation plan is given in Table 2.

**Table 2:** Process evaluation plan

**Group**	**Method**	**Aims and objectives**
Senior Health and Safety managers in entertainment/brewery organisations (n = 2)	Post-intervention semi-structured interviews	To gain information about:
		• Their perception of alcohol-related violence in the NTE and feelings of organisational responsibility
		• Existing policies and practices to address alcohol-related violence in large agencies pre-SMILE
		• Variation in organisational practice over the UK
		• Research aim 1, 5 and 6
Senior EHOs - involved in trial development and implementation (n = 3)	Post-intervention focus group	• Description of role in intervention development
		• Gain perceptions of organisational change needed to adopt SMILE
		• Description of implementation processes and integration with usual EHO practice, including barriers and facilitators
		• Research aims 1, 2, 5 and 6
EHOs (max 22) - one from each LA engaged in the delivery of the intervention	Post-intervention semi-structured interviews	• Description of role of EHOs in LPs pre-SMILE
	Routine monitoring data	• Description of practitioner participation, reception and responsiveness to the intervention
		• Process of intervention delivery, including fidelity, barriers and facilitators & extent of interagency collaboration
		• Description of location, size suitability of intervention/control premises
		• Research aims 1, 2, 3, 5 and 6.
Premises staff in 22 intervention and 22 control premises.	Post-intervention semi-structured interviews	Intervention premises:
	Routine monitoring data	• Receipt and reaction to the intervention.
		• Nature of the intervention, its acceptability and reach through organizational hierarchies, and information about how the intervention fitted with intervention premises contexts
		• Research aims 1, 4, 5, and 6.
		Control premises:
		• ‘Usual practice’ of EHO visits
		• Compare intervention EHO visits with control EHO visits
		• Research aims 1, 5 and 6

### Outcome measures

#### ***Primary outcome measure***

The primary outcome measure is number and timing of violent events between trial arms. More formally, the underlying assumption of PL interventions is that PL risks increase the likelihood of violence. However, police data are proxies to violence, recording varying aspects of the incident. One single violent incident can lead to multiple arrests and/or multiple victims and the correspondence between what is recorded and the event that produced the incident is not always clear. For example, one perpetrator who assaults two people will have committed two crimes and this is recorded as such. Therefore, a premises that registers five incidents in police data is not necessarily more risky than a premises that registers one, and under conditions of moderate rarity such biases could affect inferences particularly if there is any systematic relationship between the nature of incidents and premises type. As the primary interest is premises-level risk it is therefore reasonable to assume that these risks persist across a premises opening hours and therefore multiple incidents in one session can be assumed to partly reflect the presence of those underlying risks. In other words, we must moderate our assumptions as we can only go as far to state that the presence of one or more recorded incidents in police data suggests the presence of increased premises-level risk in a single session. We therefore assumed that one or more violent incidents indicated that for that session (defined as the period the premises was open continuously) premises-level risk was elevated. Over successive days we therefore had available data for each day and for each premises indicating whether each premises had one or more incidents on each day, or more formally whether a premises was in a state of failure. Typically, studies have previously aggregated across arbitrary time periods to assess the impact of an intervention. However, premises-related incidents might be related to specific events, such as sporting events, and would be expected to fall to zero if premises close temporarily (e.g. for refurbishment). These events are time-specific, particularly for temporary closure which is a form of censoring, and should be made explicit in any analytic strategy. While Poisson models can accommodate aggregate count data and would normally be suitable, in order to account for potential time varying covariates, censoring, multiple events and discontinuous risk intervals, the preferred approach was to use an Andersen-Gill model, a derivation of the Cox proportional hazards model used in the analysis of recurrent failure data. Longitudinal data for the twelve month period preceding trial start (April 2012 to March 2013) will be used to estimate baseline failure for inclusion in the primary analysis.

#### ***Secondary outcome measures***

##### 

**Trial arm implementation and context** The focus group, semi-structured interviews with EHOs and analysis of routine monitoring data will assess how the intervention and normal service provision operates in each LA. This will include the nature of harm reduction strategies, staffing arrangements, barriers/facilitators and integration of services. Comparative data will be drawn from semi-structured interviews conducted with the Designated Premises Supervisor (DPS) and a sample of bar staff in 44 case study premises sampled equally across intervention areas and trial arms.

##### 

**Trial arm fidelity** Interviews with EHOs, routine monitoring data and observation of the EHO training days will assess the compatibility of the intervention with existing EHO practice over Wales, the consistency with which the intervention and normal service are delivered, and how closely implementation matches the stated design and aims.

##### 

**Participation, reach and dose delivered** Interviews with EHOs and routine monitoring data will be used to assess the number and type of risks in trial premises and any patterning in levels of participation for different elements of the intervention and contamination between trial arms.

##### 

**Reception and responsiveness** Routine monitoring data will be used to explore the number and nature of risk factors identified in action plans and the number and nature of successful actions in intervention premises. Case study interviews with DPS and bar staff will explore the experiences of the intervention or normal service in terms of its acceptability, their assessment of its value to them and any barriers or facilitators to participation.

##### 

**Costs associated with the intervention** This represents the extra cost of training EHOs, the cost of EHOs delivering the intervention (time on premises plus travel), the cost of actions taken by licensees over and above what would be done as a result of a routine EHO visit and the cost of health service and criminal justice resource use generated due to violence e.g. attendances at A&E departments, hospital admissions and cost of criminal prosecutions.

### Statistical analysis

#### ***Main analysis***

The primary analysis will be ITT and will compare the two groups (n = 600) as originally randomised on the frequency and timing of violent incidents using the Andersen-Gill model [26], a derivation of the Cox proportional hazards model. This model can be used to assess the hypothesised intervention wane over the twelve month follow-up period, assess participation and dose. In addition, secondary analyses will explore the effect of the intervention on the volume of violence attributable to study premises. A per-protocol (PP) analysis will also be carried out excluding those premises in the intervention arm that did not receive the intervention. Sensitivity analyses will be carried out to determine the effect of differential ascertainment of premises closure information. Secondary analyses will also include the replacement and spare premises, however these analyses are non-randomised and provide observational estimates of intervention effect using the complete set of licensed premises (n = 837).

#### ***Qualitative analysis***

A selection of interview transcripts will be used to construct coding frameworks of dominant themes and sub-themes to form the basis of analytic framework matrices organised around the core PE evaluation research aims and any other pertinent themes that emerge from the data. All data will be confidential, and digital recordings will be stored securely and separately from transcriptions. NVivo software will be used to conduct analyses.

#### ***Economic evaluation***

The economic evaluation will be in the form of a cost effectiveness analysis from a societal perspective.

#### ***Costs***

As SMILE is additional to the usual practice received by licensed premises from statutory authorities, all costs are incremental. These include 1) EHO training: the time spent by trainers and EHOs on all training related activities will be monitored prospectively, valued using standard methods [27] and amortised over 5 years. The cost of materials, venues and any other resources used in training will also be monitored and valued using money costs incurred. 2) EHO Intervention: The time spent by EHOs in all intervention associated activities relating to each premises, including travel time, will be monitored prospectively and valued using standard methods. 3) Licensee born costs: data of the changes identified in the audits (e.g. installation of CCTV cameras, employment of additional staff, staff training) will be used to estimate the costs of the intervention to licensees. In addition, estimates of the potential cost savings to the health service and criminal justice system resulting from reductions in violent events will be made

#### ***Effects***

The unit of effectiveness for the cost effectiveness analysis will be sessions with one or more violent events, based on police records. An Andersen-Gill model will be applied to account for potential time varying covariates and censoring. The analysis will be carried out using the recurrent event model on the total number of sessions with violent events. The corresponding hazard ratio provides the percentage reduction in risk of a session involving alcohol related violence.

#### ***Cost-effectiveness analysis***

The net cost of the intervention will be assessed against the unit of effectiveness. Uncertainty will be explored through a series of one way and multivariate sensitivity analyses. Results will be reported in the form of an incremental cost effectiveness ratio (ICER) that shows the additional cost per violence-event-session averted and which can be used to demonstrate the relative cost effectiveness of SMILE versus other interventions in future evaluations. A probabilistic sensitivity analysis will be carried out using a non-parametric bootstrap method on the joint distribution of costs and effects [28]. This provides a probability value of an intervention being cost-effective within a range of willingness-to-pay (WTP) threshold values. This helps policy makers to decide whether the intervention is value for money.

## Discussion

This protocol describes a RCT of an intervention (SMILE) designed to reduce alcohol-related violence in licensed premises in all LAs across Wales. This is the first RCT of a PL intervention in the UK and has been designed to overcome a number of methodological limitations of similar studies conducted elsewhere. The potential benefits of this intervention are substantial. If the potentially low cost implementation succeeds in reducing alcohol-related violence then there will likely be substantial tangible (e.g. reducing costs to health services and the police) and intangible benefits (e.g. reducing fear of crime [29] and the psychological impact of victimisation). The results of the trial will be directly applicable to licensed premises in Wales. If the trial proves successful, then this warrants investigation further afield. Opportunities for replication will be considered for testing SMILE in England and maybe Scotland; areas that are broadly similar to Wales in regards to legislation and licensed trade. Furthermore, opportunities will be sought to generalise findings across Europe and possibly North America in areas where the on-site consumption of alcohol is a popular leisure activity and violence in the NTE is an issue.

## Abbreviations

AWLPI: All-wales licensed premises intervention; A&E: Accident and emergency; SMILE: Safety management in licensed premises; DPS: Designated premises supervisors; EHOs: Environmental health officers; ICER: Incremental cost effectiveness ratio; ITT: Intention to treat; LA: Local Authority; NHS: National health service; NISCHR: National institute for social care and health research; NTE: Night time economy; PE: Process evaluation; PL: Premises level; PP: Per-protocol; RBS: Responsible beverage server; RCI: Risk control indicator; RCT: Randomised controlled trial; RIDDOR: Reporting of injuries, diseases and dangerous occurrences regulations; RLI: Risk led intervention; SEWTU: South east wales trials unit.

## Competing interests

The authors declare they have no competing interests.

## Authors’ contributions

SCM conceived of the study. SCM, SM, VS, KH, RP, IS, JPS, LM DC and FA contributed to the original proposal from which this protocol was developed. SCM, SM, AW, and CO were responsible for the conduct of the trial with SCM as principal investigator. CO was responsible for the day to day management of the trial. AW took responsibility for and SM managed the process evaluation. CO and SCM drafted the manuscript. All authors read, contributed to and approved the final manuscript.

## Pre-publication history

The pre-publication history for this paper can be accessed here:

http://www.biomedcentral.com/1471-2458/14/21/prepub
